# Vineyard light manipulation and silicon enhance ethylene-induced anthocyanin accumulation in red table grapes

**DOI:** 10.3389/fpls.2023.1060377

**Published:** 2023-01-27

**Authors:** Maha Afifi, Alaaeldin Rezk, David Obenland, Ashraf El-kereamy

**Affiliations:** ^1^ California Table Grape Commission, Fresno, CA, United States; ^2^ Department of Botany and Plant Sciences, University of California Riverside, Riverside, CA, United States; ^3^ United States Department of Agriculture (USDA), Agricultural Research Service, San Joaquin Valley Agricultural Sciences Center, Parlier, CA, United States

**Keywords:** table grape, anthocyanin, berry color, temperature, antioxidants, hormones

## Abstract

Red color resulted from anthocyanin pigment, is an essential trait for premium table grape production. Anthocyanin biosynthesis occurs through the flavonoid pathway which includes several enzymatic reactions coded by different genes. The expression of these genes is regulated by different cultural practices, cultivars, environmental conditions, and plant hormones. Recently, we reported that the anthocyanin pathway is regulated by several factors such as light and antioxidant activity. Despite the advances in cultural practices, it is still challenging to produce table grapes with high coloration, especially under the current and expected global climate change in warmer areas such as California. In the current study, we deployed two approaches to improve the accumulation of red pigment in table grapes. The first approach involves improving the expression of critical genes involved in the anthocyanin pathway through hormonal treatments and light manipulation using a reflective ground cover (RGC). The second approach was to reduce the negative effect of heat stress through stimulation of the antioxidant pathway to help remove free radicals. Treatments included ethephon (ET) at 600 mg/L, silicon (Si) at 175 mg/L, and a commercial light-reflective white ground cover (RGC) alone and in various combinations. Treatments were conducted either with or without a combination of cluster-zone leaf removal at veraison (LR) on Flame seedless (*Vitis vinifera* L.). Data collected in 2019 and 2020 showed that the best treatment to improve berry coloration was using ET in combination with Si and RGC, applied at veraison. Adding the LR to this combination did not improve berry color any further, but rather caused a reduction in color development. RGC without conducting LR at veraison significantly increased the quantity of reflected blue and red lights as well as the red (R) to far-red (FR) ratio (R: FR) around clusters. Results were in accordance with the increase in gene expression of *flavonoid-3-O-glucosyltransferase* (*UFGT*), a key gene in the anthocyanin biosynthesis pathway, as well as *Peroxidase dismutase* (*POD*). Manipulating the light spectrum and application of silicon in combination with the ethephon treatment could be used in table grape vineyards to improve the ethylene-induced anthocyanin accumulation and coloration.

## Introduction

Red grape coloration is one of the most important traits for premium table grape production. Anthocyanin is a plant pigment that is responsible for the red color in grapes. Its synthesis is controlled by different genes regulated by several cultural practices, cultivars, environmental conditions, and plant hormones (Boss et al., 1996). Light exposure is a crucial factor that affects grape coloration through the upregulation of several genes including anthocyanin biosynthetic genes (Matus et al., 2009). Cluster coloration is correlated with the amount of light reaching the berries (Ma et al., 2019). Light is received by plant cells through photoreceptors, phytochromes, to be utilized by several plant metabolism pathways. Photosynthetically active radiation (PAR) refers to the range of solar radiation (400-700 nm) that plants can use to create energy through photosynthesis. The white light spectrum consists of several wavelengths ranging from 380 nm to 780 nm; each wavelength is received by plant cell phytochromes and provides a unique function and purpose (Demotes-Mainard et al., 2016). For example, while exposure of grape berries to blue light by covering the greenhouse with blue plastic film improves berry anthocyanin content, orange, red, and green light have no effect on berry anthocyanin content (Cheng et al., 2015). Additionally, red light was reported to improve anthocyanin content in cranberries (Zhou and Singh, 2002). Light affects anthocyanin accumulation by inducing the expression of genes involved in anthocyanin biosynthesis. Far-red light falls outside the PAR range with a wavelength of 700-850 nm; plants are sensitive to the difference between red and far-red light (Afifi and Swanton, 2012). The ratio between red and far-red light is a significant factor in inducing and accumulating anthocyanin content in several plant species; lower R:FR ratio is associated with lower anthocyanin content in corn (Afifi and Swanton, 2012), and *Stellaria longipes* (Alokam et al., 2002).

Further, it has been established that gene expression of anthocyanin biosynthesis is affected by temperature, where moderate temperature induces anthocyanin biosynthesis gene expression and high temperature represses it (Mori et al., 2005). The negative effect of the higher-than-normal temperature is becoming more obvious due to climate change and the rise in global temperature in several areas of the world (Cook and Wolkovich, 2016). To improve table grape coloration in warmer areas such as California, an ongoing research project has been conducted including the current study. The research program consisted of two parts. The first part was to identify potential plant regulatory factors associated with the expression of several genes involved in anthocyanin biosynthesis in table grapes. This part was conducted in 2016 and 2017 and showed that the lack of red color development could partially be due to low expression of anthocyanin genes and/or the accumulation of free radicals under stress conditions, which resulted in accelerated anthocyanin degradation and impaired anthocyanin biosynthesis (Afifi et al., 2021). Ethylene and abscisic acid are reported to play a major role in promoting ripening and red color development in grapes (Xu et al., 2018). Data obtained from our previous study (Afifi et al., 2021) confirmed the importance of using ethephon (as a source of ethylene) or ProTone (as a source of abscisic acid) in the vineyard to improve the red color. Thus in 2019 and in the second part of this research project two approaches were investigated to enhance the accumulation of red pigment in grapes. The first approach was to improve the expression of critical genes involved in the anthocyanin pathway through hormonal treatments or by using a light-reflective white ground cover (RGC) that has been reported to modify the light spectrum around clusters. The second approach was to activate the antioxidant system and reduce the negative effect caused by environmental stress using silicon (Si).

In response to abiotic stress, plant cells produce highly toxic reactive oxygen species (ROS) which act as a signaling molecule at low concentration and cause damage to the cell at high concentration (Hansen et al., 2002; Gill and Tuteja, 2010). During abiotic stress, the plant cell activates the enzymatic antioxidant system to remove ROS from the cell; one group of these enzymes are the peroxidases (PODs) (Foyer and Noctor, 2005). We previously reported the induction of POD in grapes in response to high temperature and it was associated with a higher anthocyanin content (Afifi et al., 2021). In addition, peroxidase activity was increased by exogenous ethylene application in *Pisum sativum*, and it seems that ethylene regulates the POD activity at the translational rather than a transcriptional level (Ridge and Osborne, 1970). Exogenous Silicon (Si) was reported to play a significant role in alleviating the abiotic stress in different plant species and consequently improved its productivity (Torabi et al., 2015, Zhang et al., 2018b). Silicon was reported to have a role in regulating ROS generation in plant tissues by triggering and activate the antioxidant system, especially under abiotic stress condition (Kim et al., 2017). Furthermore, application of exogenous silicon increased the antioxidant enzyme activity and reduced the lipid peroxidation in the salt-stressed barley plants (Liang et al., 2003). In addition to the direct effect of silicon on the antioxidant activity and ROS scavenging, Si was also reported to regulate abscisic acid (ABA), sulfur metabolism and reduce hydrogen peroxide content in barley under nutrient deficiency conditions (Maillard et al., 2018).

The whole globe is experiencing an increase in temperature, and it is anticipated that the temperature will increase by 2-5°C by 2050 (IPCC, 2022). Such an increase in temperature could negatively affect grape coloration by influencing anthocyanin biosynthesis and degradation (Mori et al., 2007). Although researchers are providing some solutions, there are more environmental factors that act against this effort and negatively impact grape coloration. Manipulation of the light spectrum in orchards has been reported to improve sugar and red color accumulation in apple fruits (Iglesias and Alegre, 2009). In addition to the significant effect on canopy management on cluster quality and health, it plays an essential role in allowing the light to reach the clusters and increasing its intensity on berries throughout ripening. Leaf removal is a common practice in table grape vineyard to reduce leaves overlapped and increase the airflow inside the vines and reduce shading (Cataldo et al., 2021). The previously obtained data led us to the hypothesis that increasing the expression of biosynthesis pathway genes and reducing oxidative stress by enhancing the activity of antioxidant enzymes might have better results than using one only approach. Therefore, the current study was conducted to investigate the impact of cluster-zone leaf removal on the efficacy of using RGC and/or Si to ameliorate the effects of ET in improving red color accumulation in grapes.

## Materials and methods

### Site selection and treatments

This study was conducted during the 2019 and 2020 seasons using Flame Seedless, as an early-season variety, grown in a commercial vineyard in Kern County, San Joaquin Valley of California. Flame Seedless vines were mature (7 years old), grafted on Harmony rootstock, and grown in loamy, sandy soil. Vines were trained on an open gable trellis system and pruned on a quadrilateral cordon, spur pruning around 60 buds per vine. Spacing at this site was 1.83 X 3.66 meters and the average number of clusters was 35 per vine. Row orientation was East/West with 95 vines per row. Monthly climate data reported by CIMIS system (https://cimis.water.ca.gov) at the experiment location in the 2019 and 2020 growing seasons is presented in **Table 1S**. At this site, Si (Silicon 7%, BAICOR, Utah, USA) at the rate of 175 mg/L active ingredient with a spray volume of 935 liters per hectare, ET (ethephon 2S L, ADAMA, NC, USA) at 600 mg/L active ingredient with a spray volume of 935 per hectare, and a six-foot wide commercial RGC (Greenhouse Megastore, CA, USA) were tested alone and in various combinations. In 2018, we studied the effect of several treatments on berry coloration including the Silicon alone and the above-mentioned treatments were selected and conducted either with or without cluster-zone leaf removal (LR) (**Table 1**). Treatments were carried out in four adjacent rows which did not receive any other practice that could induce color. In these rows, treatments started at veraison when 30% of berries in the clusters were turned red (mid-June). Data collection and berry sampling were at harvest, three weeks after starting the treatments (early July). Harvest time was determined based on the percentage of the marketable clusters that have a good color and brix of 16. In both seasons, there were twelve treatments with four replications. Each replicate contained seven vines and all measurements and sample collections were conducted from the middle five vines.

**Table 1:** Different treatments used in San Joaquin Valley.

Treatments	Concentration
Untreated clusters (control)	---
LR	---
RGC	---
RGC+LR	---
ET	600 mg/L
ET+LR	600 mg/L
ET+Si	ET (600 mg/L) + Si (175 mg/L
ET+Si+LR	ET (600 mg/L) + Si (175 mg/L)
ET+RGC	ET (600 mg/L)
ET+RGC+LR	ET (600 mg/L)
ET+Si+RGC	ET (600 mg/L) + Si (175 mg/L)
ET+Si+RGC+LR	ET (600 mg/L) + Si (175 mg/L)

Treatments were: ethephon (ET), silicon 7% (Si), light-reflective ground cover (RGC), and their combinations. Treatments have been conducted with or without cluster-zone leaf removal (LR). The concentrations of each mixture are noted in the Table; spray volume was 937 liter per hectare in all treatments.

**Figure 1:**
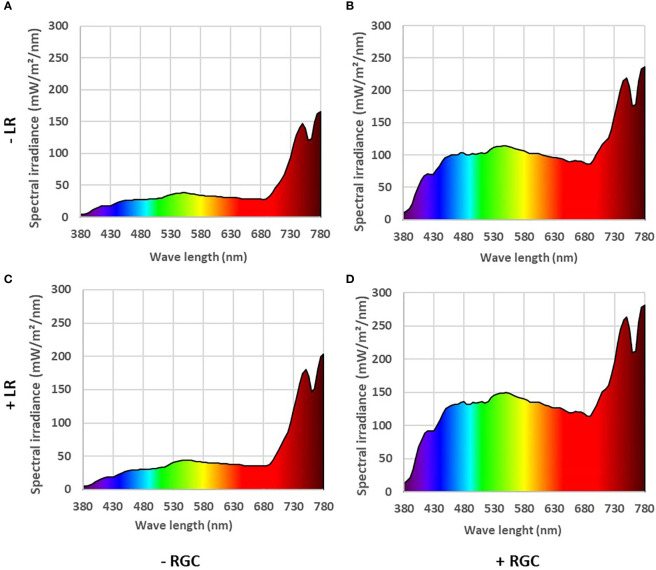
The reflected light spectrum measured at the cluster zone in 2019 as affected by using (+) **(B, D)** or without using (-) **(A, C)** light-reflective ground cover (RGC). Treatments have been conducted with (+) **(C, D)** or without (-) **(A, B)** cluster-zone leaf removal (LR). Values are the mean of four readings. Data were recorded once at the mid-day of June 17th, 2019.

In this study, chemicals were sprayed directly on the clusters using a gas-powered, hand-held wand sprayer, with a spray volume adjusted to 935 liter/hectare. The white RGC used in this study is a woven polypropylene material that is UV stabilized. This ground cover is reusable and resistant to tearing, puncturing, and weed penetration. It is also permeable, allowing water and nutrients to pass through. The six-foot wide ground cover was rolled out at veraison from both sides of a seven-vine plot near the trunk (**Figure 1S**). To avoid any effect of the RGC on the other treatments, all treatments that contained RGC were placed in the west half of the four rows. After harvest time, ground covers used were removed and reused in the second year.

### Reflected light spectrum and berry temperature measurements

The reflected light from the ground at the cluster zone was measured underneath the canopy using Gossen, MAVOSPEC BASE Industrial light meter – a miniaturized spectrometer for measuring light (Foto Und Lichtmesstechnik, GmbH, Nuremberg, Germany). This light meter measures each individual wavelength intensity over a large spectral range of 380 to 780 nm. This measurement was conducted from both north and south sides of the row and the average value was calculated. The measurement was taken by holding the instrument with the hand within the cluster zone area with the lens facing the ground. Further, the temperature of 25 random berries from each side of the row was measured in each replicate. Berry temperature was measured using a Flir, TG54-NIST non-contact thermometer with a laser pointer (Wilmington Instrument Co, CA, USA). Measurements were carried out at mid-day and the thermometer was placed six inches away from the outer berry surface. Data were recorded once at the mid-day of June 17^th^, 2019, and June 19^th^, 2020.

### Cluster color segregation

The color development of both sides of all clusters in the middle five vines of each treatment was determined visually using the color segregation method (Afifi et al., 2021). The clusters were classified into four grades: Grade A: clusters with less than 25% of colored berries; Grade B: clusters with 25-50% of colored berries; Grade C: clusters with 50-75% of colored berries; and Grade D: clusters with over 75% colored berries.

### Berry color and compositional analysis

At harvest time, fifty berries from each replicate with a total of two hundred berries per treatment were collected randomly from clusters located in the inner, central, and outer portions of the canopy. Berries were transported immediately to the laboratory on ice within two hours and then, the skin of these berries was removed and frozen immediately and stored at -80°C to extract and determine the total berry skin anthocyanin content and perform the enzyme activity. The frozen skin samples were ground using a ball mill MM300 (Retch) in the presence of liquid nitrogen. One gram of frozen ground samples was extracted into 5 mL of methanol: HCl (100:1). This extraction was used to determine total anthocyanin content spectrophotometry at 520 nm as described by Boss et al. (1996). The spectrophotometer reading was used to calculate the anthocyanin content as described by Koteski and Mojsov, 2019. Another set of fifty berries per replicate were collected to determine berry weight, length, and diameter. These berries were used to visually determine color using the color segregation method. Therefore, berries were separated into four categories: Grade A: berries with 25% color or less; B: berries with 25-50% color; C: berries with 50-75% color, and D: berries with 75-100% red color (Afifi et al., 2021). The overall color of each treatment was determined by referring to the category under which 50% of berries or more were found. In addition, the color of these berries was measured using a Konica Minolta colorimeter (CR-400; Minolta, Ramsey, N.J.) to obtain the hue angle which refers to the main berry color. Three equidistant measurements were made around the equators of each berry. Hue angle (h°) refers to the color wheel and is measured in angles; green, yellow, and red correspond to 180, 90, and 0°, respectively (McGuire, 1992; Carreño et al., 1995; Lancaster et al., 1997). Berry firmness was also measured in these berries using a FirmTech II small fruit firmness analyzer (BioWorks, Wamego, KS, USA). The Firmtech gently squeezes the berry to determine the firmness. When a berry is squeezed by the instrument load cell the force increases. The rate at which the force (grams) increases per unit of deformation (mm) is defined as firmness (grams/mm). Then these berries were macerated in an electric blender, filtered through a paper towel, and an aliquot of juice was used to determine soluble solids (°CBrix), pH, and titratable acidity (TA). Soluble solids were determined using a tabletop Milwaukee MA871-BOX digital refractometer (Milwaukee Instruments, Inc., NC, USA). The TA and pH were determined by titrating a 40 mL aliquot of juice with 0.1 N NaOH to a pH of 8.2 using an automatic titrator Excellence T5 (Mettler-Toledo, OH, USA).

### Quantitative real-time PCR

Total RNA was isolated from frozen berry skin samples per the protocol described by Boss et al. (1996). DNA was removed from samples using RNase-free RQI treatment per the manufacturer’s instructions (Promega, Madison, WI, USA) followed by a cleanup with the RNeasy mini kit (Qiagen, Valencia, CA, USA). cDNA was synthesized from total RNA using the qScript™ cDNA Synthesis kit (Quanta Biosciences, MD, USA). Quantitative real-time expression was performed using PerfeCTa SYBR Green SuperMix ROX (Quanta Biosciences, MD, USA) on the ABI7300 real-time system (Applied Biosystems, CA, USA) and using *UDP-glucose: flavonoid-3-O-glucosyltransferase* (*UFGT*) gene’s primer designed with the Primer Express 2.0 software (Applied Biosystems) as described in (Afifi et al., 2021). Relative quantification for UFGT gene was calculated by the 2–ΔΔT method (Livak and Schmittgen, 2001) using the grape ubiquitin gene as a constitutive control (Fujita et al., 2005). The ubiquitin primer was designed from a tentative consensus sequence TC38636 and described in (Afifi et al., 2021). QRT-PCR analyses were performed using three biological replicates using sets of cDNA from independent samples. Gene expression was calculated as an increase/decrease fold change in reference to the sample collected from the untreated treatment.

### Polyphenol oxidase enzyme assay

Frozen 0.5 g of berry skin collected at harvest was extracted in 5 mL of 50 mM sodium phosphate buffer (pH 7.0) containing 1 mM ethylenediaminetetraacetic acid (EDTA) and 5% soluble polyvinyl pyrrolidone (Sigma-Aldrich, St. Louis, MO, USA). The resulting homogenate was centrifuged at 3,000g for 30 min at 4°C and the supernatant was utilized for subsequent measurement of POD activity.

The POD activity was assayed as described by Troiani et al. (2003), one hundred microliter aliquot of tissue extracts was added to 3 mL of assay solution, consisting of 13 mM guaiacol (Amresco, OH, USA), 5 mM H_2_O_2_, and 50 mM sodium phosphate buffer (pH 7.0) (Cheng et al., 2013). Increases in optical density were then measured at 470 nm for 3 min at 25°C. POD activity was expressed as the change in absorbance per minute per gram of fresh skin weight.

### Experimental design and statistical analysis

Treatments were conducted in a randomized complete block design for both seasons. Each block consists of one row that contains all randomly distributed treatments. One way ANOVA statistical analyses were performed using SigmaStat (Systat, CA, USA). The significant difference between treatments was evaluated using Tukey’s Honestly Significant Difference (HSD) multiple comparison test (*p* ≤ 0.05). The clusters and berries percentage data were not statistically analyzed.

## Results

### Light quality surrounds the clusters and berry temperature

**Figure 2:**
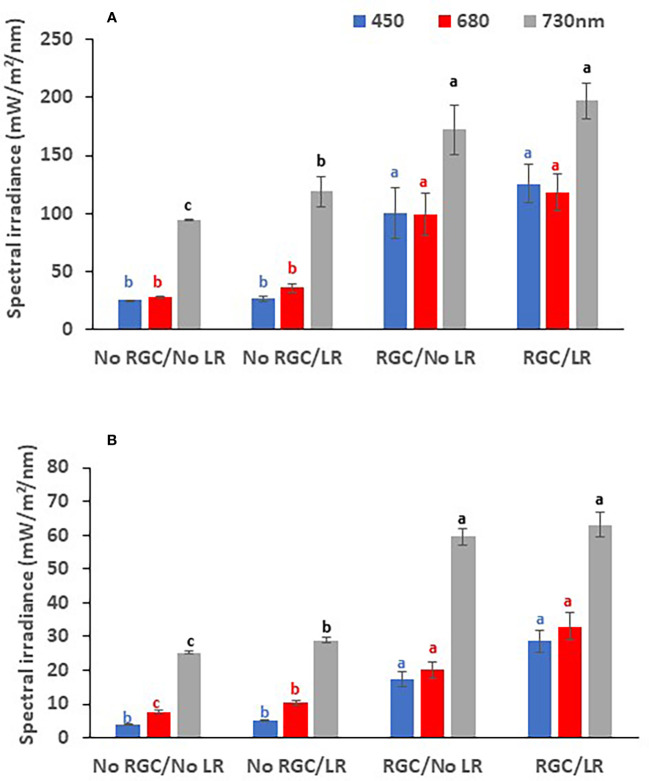
The quantity of reflected blue (at 450 nm), red (at 680 nm), and far-red (at 730 nm) lights measured at the cluster zone as affected by using light-reflective ground cover (RGC) and/or conducting the cluster-zone leaf removal (LR) in 2019 **(A)** and 2020 **(B)**. Values are the mean of four readings, and the error bars represent the standard deviation. Different colored letters on bars indicate significant differences in same light wavelength at p < 0.05 according to the Tukey HSD test within the same year. Data were recorded once at the mid-day of June 17^th^, 2019, and June 19^th^, 2020.

In both years of the study, data confirmed that using the light-reflective ground cover (RGC) without conducting the cluster-zone leaf removal (LR) significantly increased the quantity of reflected blue (at 450 nm), red (at 680 nm), and far-red (at 730 nm) lights surrounding the clusters compared to the uncovered ground (**Figures 1**, **2**). Further, RGC increased the red (R) to far-red (FR) ratio (R:FR) surrounding the clusters (**Table 2**). Data also revealed that the cluster-zone leaf removal alone had no significant effect on the reflected blue light surrounding the clusters. The effect of the cluster-zone leaf removal alone (LR) on R:FR had no constant trend and overall no statistical difference in the R:FR was observed (**Table 2**). The values of the reflected blue, red, and far-red lights from the uncovered ground treatments with no leaf removal (-RGC/-LR) were 25, 28, and 95 mW/m²/nm in 2019 and 4, 8, and 25 mW/m²/nm in 2020. These values increased significantly by using the RGC/-LR to 101, 99, and 186 mW/m²/nm in 2019 and 5,10 and 29 mW/m²/nm in 2020. Adding the cluster-zone leaf removal to the RGC (+RGC/+LR) caused a slight increase in these values to reach 126, 133, and 197 mW/m²/nm in 2019 and 29, 33 and 63 mW/m²/nm in 2020 (**Figure 2**). We noticed that light measurements in 2020 is lower than the 2019 data and this could be due to the use of the previous year ground cover and the difference in canopy management that allowed more light reflection in 2019 compared to 2020 season. Further, the RGC increased the R:FR to 0.57 and 0.55 compared to 0.30 and 0.30 in the uncovered ground in 2019 and 2020, respectively (**Table 2**). Adding the cluster-zone leaf removal to the RGC did not cause any significant increase in this ratio; values were 0.59 and 0.50 in 2019 and 2020, respectively (**Table 2**).

**Table 2:** The reflected red to far-red ratio (R:FR) measured at the cluster zone as affected by using (+) or without using (-) light-reflective ground cover (RGC).

	2019		2020		
	-RGC	+RGC	-RGC	+RGC	645
-LR	0.30b	0.57a -	0.30B	0.55A	646
+LR	0.30b	0.59a	0.36B	0.50A	647

Treatments have been conducted with (+) or without (-) cluster-zone leaf removal (LR) in 2019 and 2020 seasons. Values are the mean of four replicates. Different letters indicate significant differences within the same year at p < 0.05 according to the Tukey HSD test within the same season. Different lower-case letters indicate a significant difference in 2019 while capital letters are used for 2020.

### Flame seedless color as affected by the different treatments

#### Cluster color segregation

**Figure 3:**
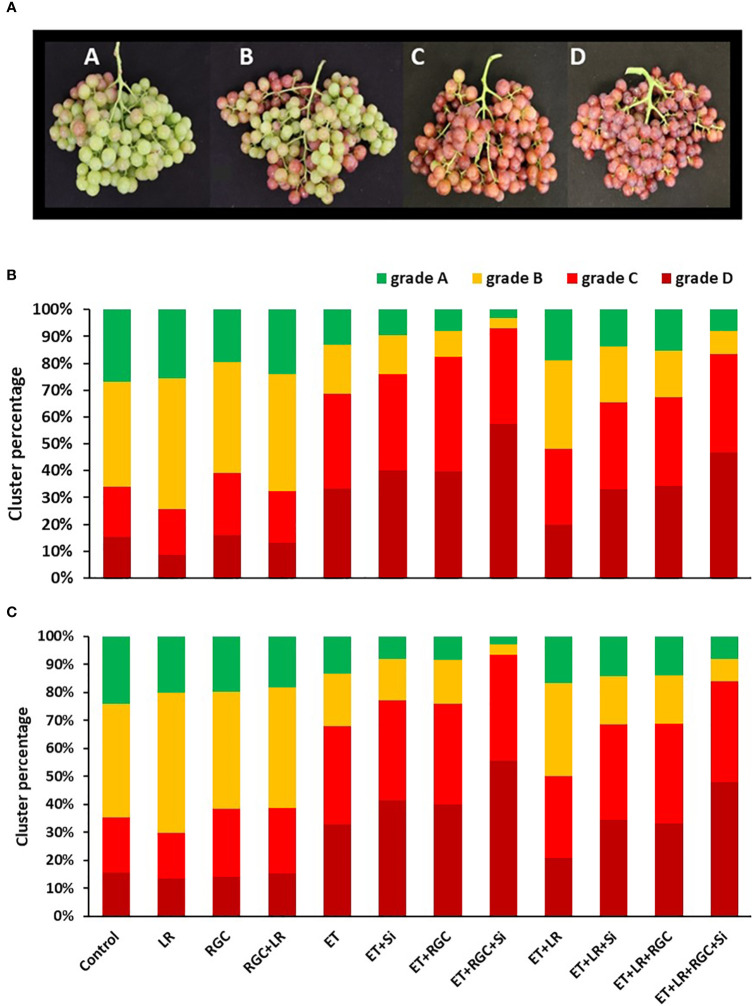
Flame Seedless cluster color segregation as affected by the different treatments in 2019 **(B)** and 2020 **(C)**. Treatments were: ethephon (ET), silicon 7% (Si), light-reflective ground cover (RGC), and their combinations. Treatments have been conducted with or without cluster-zone leaf removal (LR). Different grades used to evaluate Flame Seedless clusters color visually at harvest time. Grades are: A: clusters with less than 25%; B: clusters with 25-50%; C: clusters with 50-75%; and D: clusters with over 75% red-colored berries **(A)**.

Data revealed that the best treatment to improve cluster coloration was using ethephon in combination with silicon and the light-reflective ground cover (**Figure 3**). Adding the cluster-zone leaf removal treatment to this last combination treatment caused a reduction in the cluster color development.

For example, in 2019, the total number of clusters that have 50 to over 75% red color berries (grades C and D) increased from 34% in the untreated treatment to 69% by using the ethephon alone. This number increased to 76% by using ethephon in combination with silicon and to 83% by using ethephon in combination with RGC (**Figure 3A**). Using ethephon in combination with silicon and the RGC (ET+Si+RGC) increased the percentage of the clusters in grades C and D to 93% compared to 69% by using ethephon alone. Adding the cluster-zone leaf removal (LR) to the ethephon in combination with silicon and the RGC (ET+Si+RGC+LR) decreased the percentage of clusters in grades C and D to 84%. This data indicates that ethephon in combination with silicon and the RGC (ET+Si+RGC) increased the total number of clusters in grades C and D by 24% when compared to using ethephon alone (ET). The same trend was observed during the 2020 season (**Figure 3B**). We did not observe any sun burn or cluster damage as a result of the ground cover.

#### Berry color

**Figure 4:**
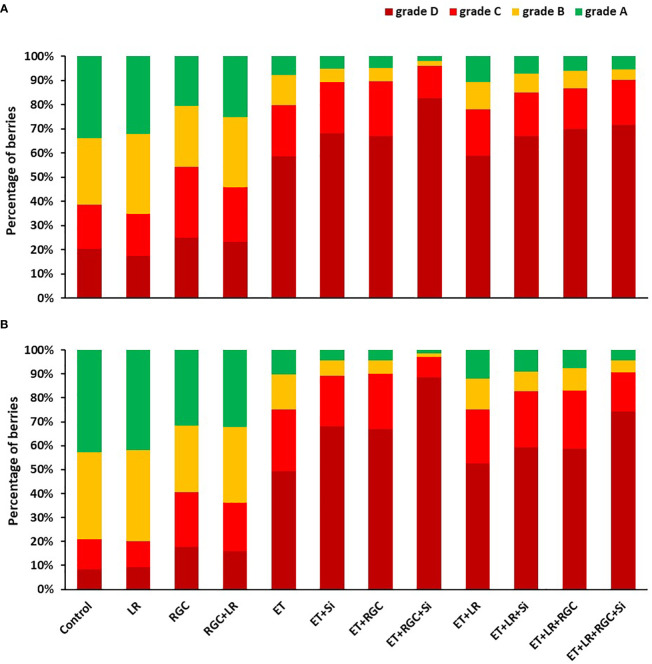
Percentage of Flame Seedless berries that fall within the different color categories as affected by the different treatments in 2019 **(A)** and 2020 **(B)**. Treatments were: ethephon (ET), silicon 7% (Si), light-reflective ground cover (RGC), and their combinations. Treatments have been conducted with or without cluster-zone leaf removal (LR). The different four color categories are: Grade A: berries with 25% color or less; B: berries with 25-50% color; C: berries with 50-75% color, and D: berries with 75-100% red color.

The color segregation evaluation for berries at harvest is presented in **Figure 4**. Data showed that the highest percentage of berries found under categories C and D combined (50-75% and 75-100% red color) were collected from the combined treatment of ethephon, silicon and the reflective ground cover (ET+Si+RGC). The percentage of the berries under these two categories increased from 35% and 25% to 95% and 97% in 2019 and 2020, respectively (**Figures 4A, B**). Adding the cluster-zone leaf removal to this last combination treatment caused a reduction in the berries’ color, however, the values (88% and 90%) are still higher than the control for these two above mentioned categories.

**Figure 5:**
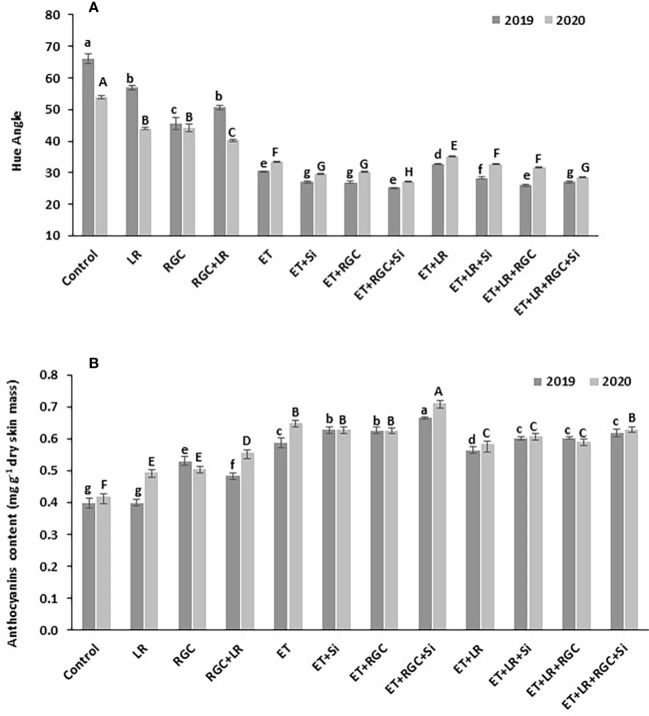
Average of hue angle for Flame Seedless grapes **(A)** and anthocyanin content **(B)** as affected by the different treatments. Treatments were: ethephon (ET), silicon 7% (Si), light-reflective ground cover (RGC), and their combinations. Treatments have been conducted with or without cluster-zone leaf removal (LR). Values are the mean of four readings, and the error bars represent the standard deviation. Different letters on bars indicate significant differences within the same year at *p* < 0.05 according to the Tukey HSD test within the same season. Different lower-case letters indicate a significant difference in 2019 while capital letters are used for 2020.

This data was also confirmed by measuring the hue angle (h) of the Flame Seedless berries at harvest time (**Figure 5A**). The hue angle is negatively correlated with the red color of berry skin, which means a lower hue value reflects darker red color in berry skin (Peppi et al., 2006). In both years, the hue angle (h) was significantly lower in grapes that were treated with ethephon in combination with silicon and the light-reflective ground cover (ET+Si+RGC) compared to other treatments. Lower hue angle values indicate that grapes in this treatment were darker in red color than other treatments (**Figure 5A**). This value increased significantly by adding the cluster-zone leaf removal to this treatment (ET+Si+RGC+LR), indicating a reduction in the berry red color (**Figure 5**). Together, these findings confirmed the data obtained from the cluster color segregation. A represented picture of berries from different treatments from 2019 and 2020 seasons is presented in (**Figures 3S**, **4S**).

### Spectrophotometric analysis of total anthocyanin content

The total berry skin anthocyanin content of the different treatments at harvest is presented in **Figure 5B**. In 2019, no significant difference in total anthocyanin content was observed between the untreated berries with or without cluster-zone leaf removal. However, leaf removal alone increased berry anthocyanin during 2020 season (**Figure 5B**). A significant increase in total anthocyanin content was observed by using the RGC; however, this increase was not significantly chnaged by adding cluster-zone leaf removal to this treatment. Data also revealed that ethephon in combination with silicon and the light-reflective ground cover (ET+Si+RGC) increased total anthocyanin content significantly compared to other treatments. Adding the cluster-zone leaf removal treatment to this combination (ET+Si+RGC+LR) significantly reduced the total berry skin anthocyanin content compared to the (ET+Si+RGC) treatment.

### Berry composition as affected by the different treatments

**Figure 6:**
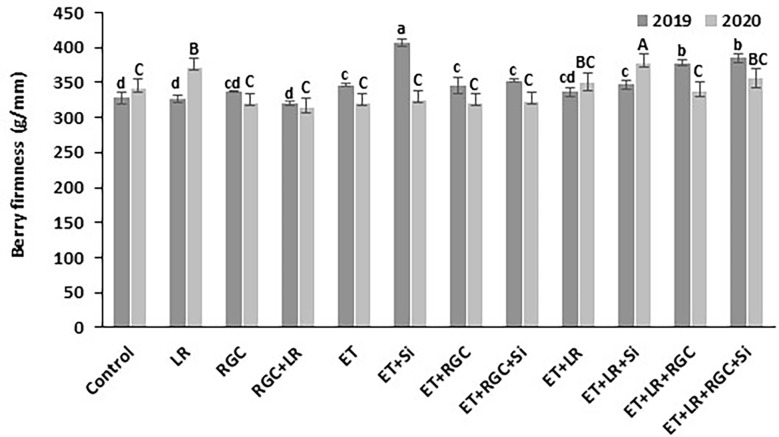
Berry firmness as affected by the different treatments. Treatments were: ethephon (ET), silicon 7% (Si), light-reflective ground cover (RGC), and their combinations. Treatments have been conducted with or without cluster-zone leaf removal (LR). The effects of Si is not reported in the figure. Values are the mean of four readings, and the error bars represent the standard deviation. Different letters on bars indicate significant differences within the same year at *p* < 0.05 according to the Tukey HSD test within the same season.

In both years, data revealed that different treatments did not influence berry weight and dimensions of the Flame Seedless grapes (**Table 3**). However, a significant increase in berry length was observed when using ethephon with silicon and the light-reflective ground cover (ET+Si+RGC) compared to the untreated berries only during 2019 season (**Table 3**). There were no significant differences in sugar content (°CBrix) and pH of Flame Seedless grapes across treatments. However, using ethephon alone or in combination with any other treatment significantly reduced berry titratable acidity (TA) in 2019 season, this effect was not obvious during 2020 season (**Table 3**). The difference response of the berry acidity to the ethephon treatment could be due the variation in the average temperature between the two seasons especially during the month of May (**Table S1**). Berry acidity is very dependent on temperature (Fowles, 1992). Overall, berry firmness did not change significantly following any treatments, except for in 2019, a significant increase was observed when silicon and ethephon were used in combination (**Figure 6**).

**Table 3:** Berry quality paraments as affected by the different treatments.

	Berry Weight (g)	Berry Diameter (mm)	Berry Length (mm)	Berry TSS (%)	Berry TA (%)	Berry pH
2019						
Control	6.41a	21.48a	22.04ab	15.08abc	0.59a	3.43g
LR	6.50a	21.34a	21.80b	15.15abc	0.62a	3.45fg
RGC	6.42a	21.76a	22.48ab	15.23abc	0.62a	3.57a
RGC+LR	6.10a	21.31a	22.31ab	14.78c	0.62a	3.47efg
ET	6.39a	21.55a	22.13ab	14.93bc	0.53a	3.50cdef
ET+Si	6.48a	21.83a	22.31ab	14.85bc	0.54b	3.51bcde
ET+RGC	6.13a	21.32a	22.19ab	15.18abc	0.54b	3.55abc
ET+RGC+Si	6.56a	21.87a	22.67a	15.05abc	0.55b	3.52bcd
ET+LR	6.37a	21.51a	22.36ab	15.33ab	0.53b	3.49def
ET+LR+Si	6.17a	21.78a	22.24ab	14.95abc	0.52b	3.49def
ET+LR+RGC	6.37a	21.87a	22.05ab	15.00abc	0.55b	3.54abc
ET+LR+RGC+Si	6.32a	21.74a	22.31ab	15.45a	0.52b	3.55ab
2020						
Control	6.07a	21.55ab	21.97a	13.70c	0.66abc	3.41cd
LR	5.87ab	21.41abc	21.50ab	13.98bc	0.67ab	3.44bcd
RGC	5.81ab	21.34abc	21.61ab	14.08abc	0.63bc	3.46abc
RGC+LR	5.87ab	21.48abc	21.60ab	14.38abc	0.65abc	3.45abc
ET	5.88ab	21.45abc	21.69ab	14.70ab	0.62bc	3.50ab
ET+Si	6.01ab	21.61a	21.96a	14.63ab	0.67ab	3.48abc
ET+RGC	5.74ab	21.30abc	21.35ab	14.05abc	0.66abc	3.43cd
ET+RGC+Si	5.92ab	21.39abc	21.70ab	14.20abc	0.66abc	3.43cd
ET+LR	5.62ab	21.05c	21.43ab	14.33abc	0.62c	3.45ab
ET+LR+Si	5.86ab	21.47abc	21.71ab	14.90a	0.62c	3.51a
ET+LR+RGC	5.60b	21.18abc	21.19b	13.60c	0.69a	3.39d
ET+LR+RGC+Si	5.72ab	21.11bc	21.26ab	13.68c	0.68a	3.42cd

Treatments were: ethephon (ET), silicon 7% (Si), light-reflective ground cover (RGC), and their combinations. Treatments have been conducted with or without cluster-zone leaf removal (LR). Values are the mean of four replicates. Different letters indicate significant differences within the same year at p < 0.05 according to the Tukey HSD test within the same season.

### Berry temperature

**Figure 7:**
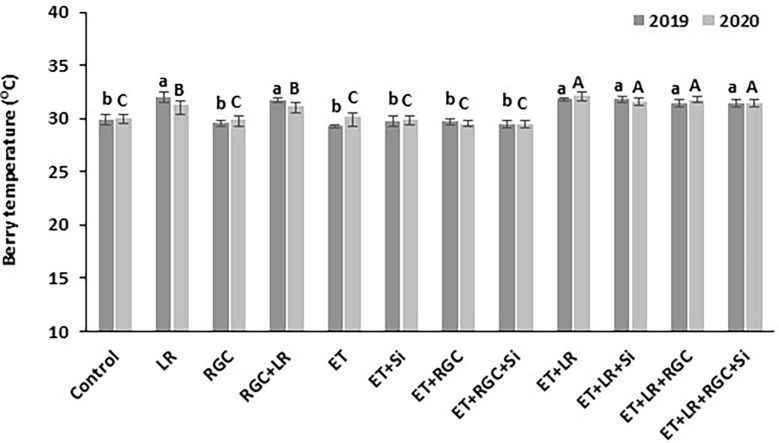
Berry temperature measured at mid-day as affected by the different treatments. Treatments were: ethephon (ET), silicon 7% (Si), light-reflective ground cover (RGC), and their combinations. Treatments have been conducted with or without cluster-zone leaf removal (LR). The effect of Si alone is not reported in the figure, Values are the mean of four readings, and the error bars represent the standard deviation. Different letters on bars indicate significant differences within the same year at *p* < 0.05 according to the Tukey HSD test within the same season. Different lower-case letters indicate a significant difference in 2019 while capital letters are used for 2020. Data were recorded once at the mid-day of June 17^th^, 2019, and June 19^th^, 2020.

Our data showed that the white reflective ground cover used in this study did not increase berry temperature (**Figure 7**). On the other hand, adding cluster-zone leaf removal to the different treatments caused a significant increase in berry temperature by 2-3°C.

### Quantitative real time PCR and POD enzyme activity

**Figure 8:**
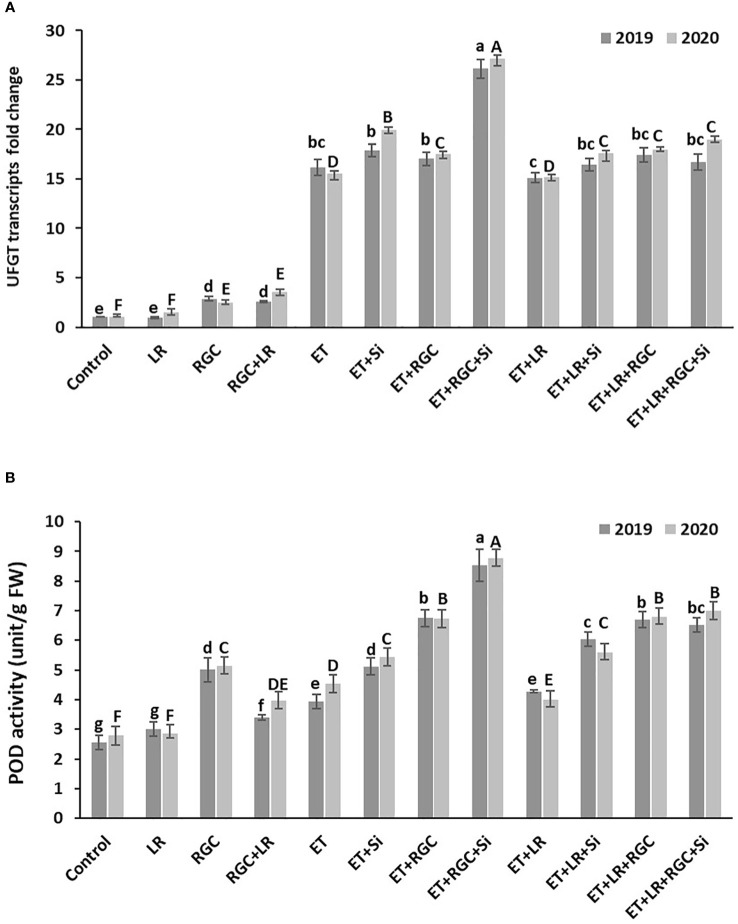
*UFGT* gene expression **(A)** and POD enzyme activity **(B)** affected by different treatments measured at one week after application. Treatments were: ethephon (ET), silicon 7% (Si), light-reflective ground cover (RGC), and their combinations. The effect of Si alone is not reported in the figure. Treatments have been conducted with or without cluster-zone leaf removal (LR). Values are the mean of four readings, and the error bars represent the standard deviation. Different letters on bars indicate significant differences within the same year at *p* < 0.05 according to the Tukey HSD test within the same season. Different lower-case letters indicate a significant difference in 2019 while capital letters are used for 2020.

Real-Time PCR data revealed that regulating light quality using white ground cover increased the expression of *UFGT*, a major gene involved in anthocyanin biosynthesis in grapes. Similar to anthocyanin content, *UFGT* expression increased by1.5 fold in the leaf removal treatment, about 2.47 fold in the RGC treatment, and fifteenfold in the ethephon treatment (**Figure 8A**). It is obvious that *UFGT* gene expression significantly improved when ethephon treatment was combined with RGC and silicon reaching around twenty-seven-fold. That increase was reduced to around seventeen-fold by adding leaf removal to this treatment. Further, POD activity showed a significant difference among treatments (**Figure 8B**), with the trend being similar to what we found in *UFGT* expression and anthocyanin content.

## Discussion

We have previously reported that the lower level of anthocyanins is associated with the lower expression of the genes involved in the anthocyanin biosynthesis pathway and the lower activity of several antioxidant enzymes (Afifi et al., 2021). The previously obtained data led us to the hypothesis that increasing the expression of biosynthesis pathway genes by exogenous treatments and reducing oxidative stress by enhancing the activity of antioxidant enzymes might have better results than using one only approach. In the current study, we demonstrated that the ground reflective plastic cover could be used with regular plant growth regulators to manipulate light quality and quantity surrounding the clusters and improve the expression of anthocyanin biosynthesis genes, which in turn, enhances the red color of table grapes. Our results showed that the ground cover increased the overall amount of light by reflecting more light from the ground towards the cluster zone. The variation in the radiative regimes between 2019 and 2020 was due to the use of the same ground reflective plastic cover used in the first year. That caused a reduction in the reflected light; however, it is still effective in improving berry coloration. The reuse the same ground cover for ore than one year will reduce the cost of the treatment. In addition, the ground cover altered the R:FR ratio in the reflected light. Low R:FR light ratio was reported to be associated with lower anthocyanin content in several plant species (Afifi and Swanton, 2012). Further, the ground cover increased the amount of blue light which is reported to have some benefits for plant development, including anthocyanin biosynthesis in grapes and strawberries (Cheng et al., 2015; Zhang et al., 2018a). We have used silicon as an agent to reduce heat stress; in this notion silicon was reported to have a significant role in reducing oxidative stress in several plant species (Torabi et al., 2015, Zhang et al., 2018 and Kim et al., 2017). Other chemicals are available to reduce heat stress such as kaolin clay (Frioni et al., 2019), however, it can not be used in table grapes due to the white color traces left on the berries after treatment.

It seems that the higher anthocyanin level we observed in the ground cover treatments could be due to its effect on modifying the light spectrum around the clusters and its association with an increase in the expression of *UFGT*, a critical gene in the anthocyanin biosynthesis pathway. Anthocyanin biosynthesis is controlled by several genes and their transcription factors which are affected by various environmental factors such as light (Zhou and Singh, 2002; Ma et al., 2019). Light is well known for its crucial role in plant photosynthesis and, therefore, its productivity. In addition, light is essential for anthocyanin accumulation and the coloration of red grapes (Guan et al., 2016). Exclusion of artificial light or sunlight reduced anthocyanin accumulation (Guan et al., 2014; Guan et al., 2016; Lee, 2017) and the expression of several genes involved in anthocyanin biosynthesis pathway (Guan et al., 2014; Ma et al., 2019). Additionally, sunlight exposure at veraison increased the expression of the MYB, a key transcription factor that controls the anthocyanin biosynthesis in grapes (Matus et al., 2009). The promoter of this gene contains a sequence responsible for the response to various factors. Several *cis* elements for light response (LRE) were identified in the promoters of the anthocyanin biosynthesis genes and transcription factors *VvMYB86* (Cheng et al., 2021), *UFGT* (Chervin et al., 2009) and *MYBA1* (Afifi et al., 2021). Possessing these *cis* elements along with other hormone-responsive elements confirms the notion that light combined with these hormones might have a synergetic effect in improving anthocyanin biosynthesis pathway.

Our data showed that the light manipulation effect was magnified by combining the ground cover with the ethephon treatment and was even better when adding silicon to the spray solution. It seems that, together, these three factors display a synergetic effect. It could be that light and ethephon improve the expression of anthocyanin genes as shown in the expression of *UFGT* (Mazza, 1995; El-Kereamy et al., 2003) while silicon acts as a stress reliever (Torabi et al., 2015, Zhang et al., 2018b) by improving the antioxidant enzyme activities and preventing anthocyanin degradation under high-temperature conditions. The positive effect of the ground cover on anthocyanin accumulation was further increased by combining this treatment with silicon spray. This treatment resulted in the highest amount of anthocyanin, a high level of *UFGT* transcript, and a high level of peroxidase activity. It was reported that the peroxidase enzyme reacts with the hydrogen peroxide present in the plant cell and converts the toxic level of ROS into water (Gulen and Eris, 2004).

It seems that our hypothesis was correct. It is suggested that growers and scientists look at improving the red coloration in table grapes from multiple points of view and consider controlling more than one factor, rather than focusing on only one treatment such as plant growth regulators. Adding the cluster-zone leaf removal to the combined treatment of ethephon, silicon and ground cover did not further improve berry color, but rather caused a reduction in color development. Light quality measurements demonstrated that the light-reflective white ground cover increased the reflected blue and red lights as well as the red to far-red ratio (R:FR) surrounding the clusters. These are key factors for enhancing anthocyanin biosynthesis in plants. Adding cluster-zone leaf removal to RGC increased R:FR; however, it caused a significant increase in berry temperature. Light and temperature are well known to influence anthocyanin accumulation in grapes. Thus, it seems that the reduction in color accumulation caused by the cluster-zone leaf removal is due to high temperature exposure. Our finding is in harmony with the fact that high temperature, between 32 and 35 °C, inhibits anthocyanin accumulation in apple (Azuma et al., 2012; Wang et al., 2016). This finding opposes that of other authors who found that leaf removal increased anthocyanin content (Yue et al., 2021). The same idea was in use in California and other table grape productions around the world; however, we demonstrated that under the current situation, and in some warmer areas, leaf removal may not be beneficial for anthocyanin accumulation. However, the final outcome of the leaf removal is depending on several factors including the weather condition, the severity and the timing of the leaf removal (Jeong et al., 2004; Tarricone et al., 2020; Cataldo et al., 2021). We confirmed that leaf removal at veraison when 30% of berries in the clusters were turned red (mid-June), increased light interception at the cluster zone; however, high berry temperature can significantly suppress anthocyanin metabolism. Performing the leaf removal at different stages such as pre-bloom stage might result in different results. The final anthocyanin accumulation in fruits and berries is determined by the relation between accumulation and degradation (Liu et al., 2018). Therefore, reducing anthocyanin degradation will help fruit and berries possess more anthocyanin, resulting in a brighter red color. It is crucial to avoid increasing berry temperature when improving light interception at the cluster zone; this could be achieved by using the light-reflective white ground cover. It is well documented that anthocyanin biosynthesis is affected by high temperature and that it requires cooler nights as well as moderate day temperature (Mori et al., 2005). When these conditions are not available, the higher-than-normal temperature negatively affects anthocyanin biosynthesis causing poor grape coloration.

The negative effect of high temperature could be due to the production of free oxygen species (ROS) which act as stressors for plant growth and development, thus inhibiting anthocyanin biosynthesis and/or anthocyanin degradation (Venios et al., 2020). While the ROS signaling pathway plays an important role in plant exposure to stress, excessive amounts of ROS could be damaging to the plant metabolism which includes anthocyanin biosynthesis. Luckily, plants have a scavenging system to reduce the negative effects of ROS (Huang et al., 2019; Venios et al., 2020; Dvořák et al., 2021). Our results showed an increase in the POD activity in response to ethephon, silicon and white RGC treatments. POD is a key enzyme in the antioxidant system that removes the ROS from the plant cell. Similar to our finding, the induction of Peroxidase in response to exogenous ethylene was previously reported in Pea (Cassab et al., 1988). In addition, the antioxidant enzyme activity and anthocyanin accumulation were reported to be regulated by light *Brassica campestris ssp*. (Zhu et al., 2017). It is possible that the higher anthocyanin and better coloration observed by combining the ethephon, ground cover and silicon could be due to the multi spectrum effect on anthocyanin biosynthesis and the activation of the antioxidant enzyme activity. In 2018, we studied the effect of several treatments on berry coloration and Silicon alone caused a significant slight increase in berry anthocyanin content (Unpublish data). So, it seems that the triple treatment has a synergetic effect on improving the berry anthocyanin content and coloration.

We have also noticed larger berries in the treatment with the ground reflective cover; however, the increase was not significant. We do not exclude the possibility of other roles played by ground cover and high light quantity and quality on other berry quality parameters, as the stage in which the ground cover is applied could also be a factor to consider. It is well known that water deficit induced anthocyanin accumulation in red grapes (Afifi et al., 2021). However, the installation method of the ground cover from the two side of the row leaving around 30 cm in the middle of the row allowed the irrigation water to freely reach the rootzone and avoid any restriction on water supply. So, there is no possibility that the improve in anthocyanin documented in this study is due to the water deficit. We are in the process of testing the effect of the longer-term use of the ground cover on plant water status and fruit quality. Further study is undergoing to determine the effect of light manipulation at stages other than veraison on berry quality. The current data are essential to avoid the expected negative effects of global climate changes which have already affected grape quality in some areas of the world (Cook and Wolkovich, 2016) and opens a new avenue for using light as a sustainable treatment to improve berry quality.

## Conclusion

The poor red coloration is a significant issue in Flame seedless table grapes, especially under challenging high temperatures in several areas of table grape production worldwide. The current study showed the importance of light quantity and quality on ethylene-induced anthocyanin accumulation in red table grapes and its coloration. Manipulating light conditions surrounding the vines using a light-reflective white ground cover in addition silicon enhanced the effect of ethephon on increasing the expression of *flavonoid-3-O-glucosyltransferase* (*UFGT*), a key gene in the anthocyanin biosynthesis pathway, as well as *Peroxidase dismutase* (*POD*), an important enzyme that helps plant cell to cope with the various stress conditions. The treatment was carried out at veraison when 30% of berries in the clusters were turned red and the maximum red grape coloration was observed when combining ethephon with the ground white plastic cover and silicon due to enhancing both anthocyanin biosynthesis and antioxidant enzyme activity. Using the light-reflective white ground cover did not restrict the water supply as the water was freely reaching the vine rootzone. Leaf removal at this stage increased berry temperature that might reduce the effectiveness of ethephon on berry coloration. However, we do not exclude different effects of leaf removal when performed at different stages such ad pre-bloom.

## Data availability statement

The original contributions presented in the study are included in the article/**Supplementary Material**. Further inquiries can be directed to the corresponding author.

## Author contributions

MA performed experimental design, field work, sample collections, data analysis, and prepared the manuscript. AR performed field work, sample collections and analysis, data analysis and preparation. DO participated in experimental design and edited the manuscript. AE-K performed experimental design, data analysis, obtain funding, and edited the manuscript. All authors contributed to the article and approved the submitted version.
