# Supplementary figures and images for: Vineyard light manipulation and silicon enhance ethylene-induced anthocyanin accumulation in red table grapes
Source: Front Plant Sci. 2023 Jan 27;14:1060377. doi: 10.3389/fpls.2023.1060377 (PMC9911529; doi:10.3389/fpls.2023.1060377)

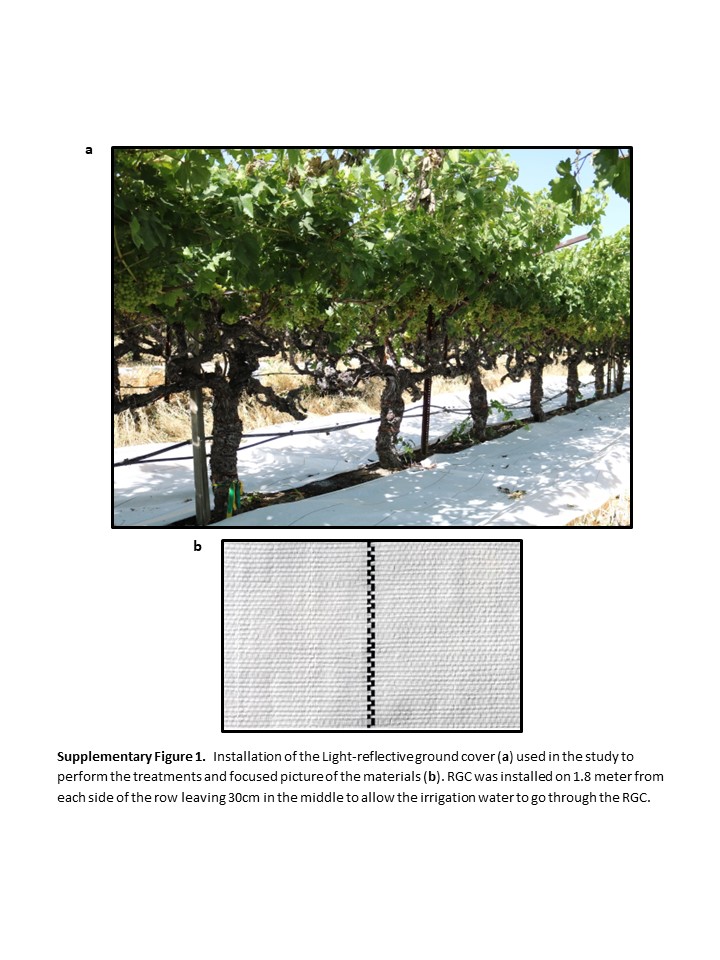

Supplement: Supplementary file 1 [file Image_1.jpeg]

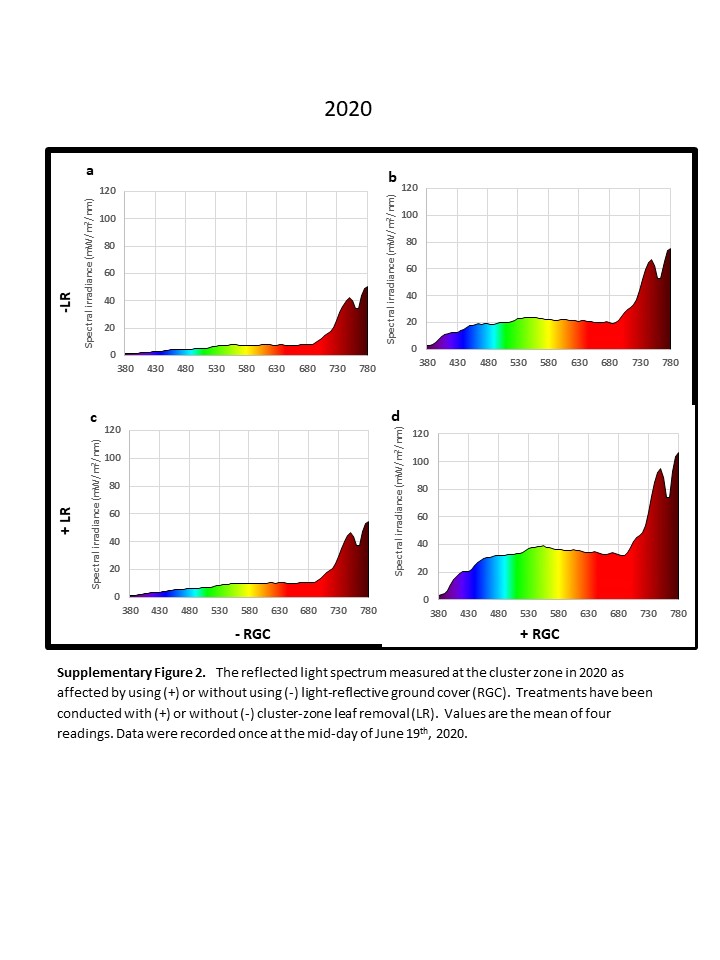

Supplement: Supplementary file 2 [file Image_2.jpeg]

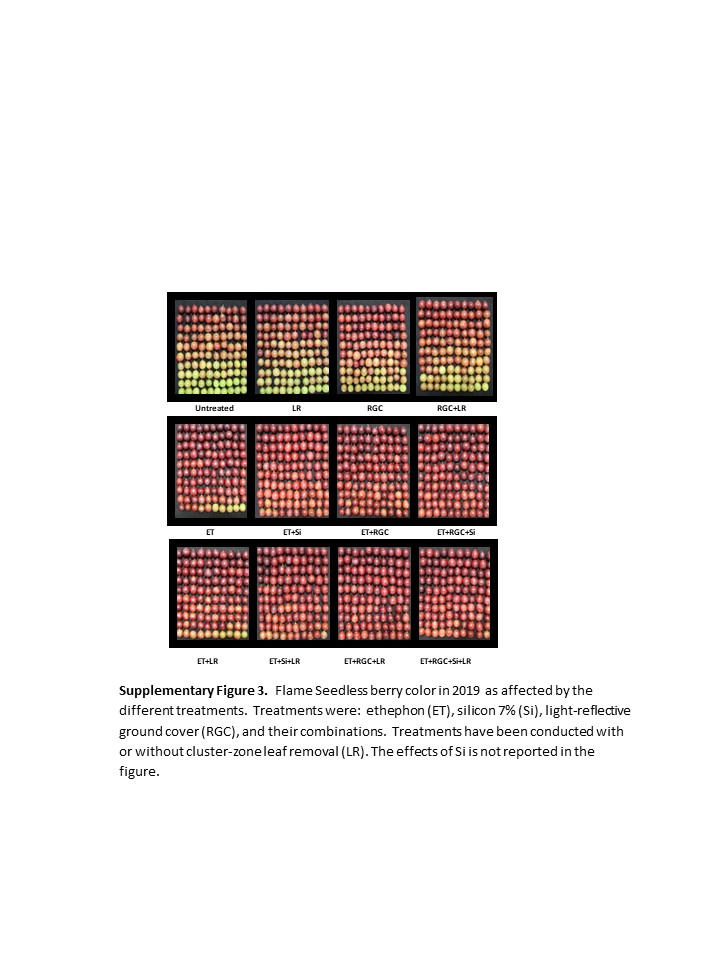

Supplement: Supplementary file 3 [file Image_3.jpeg]

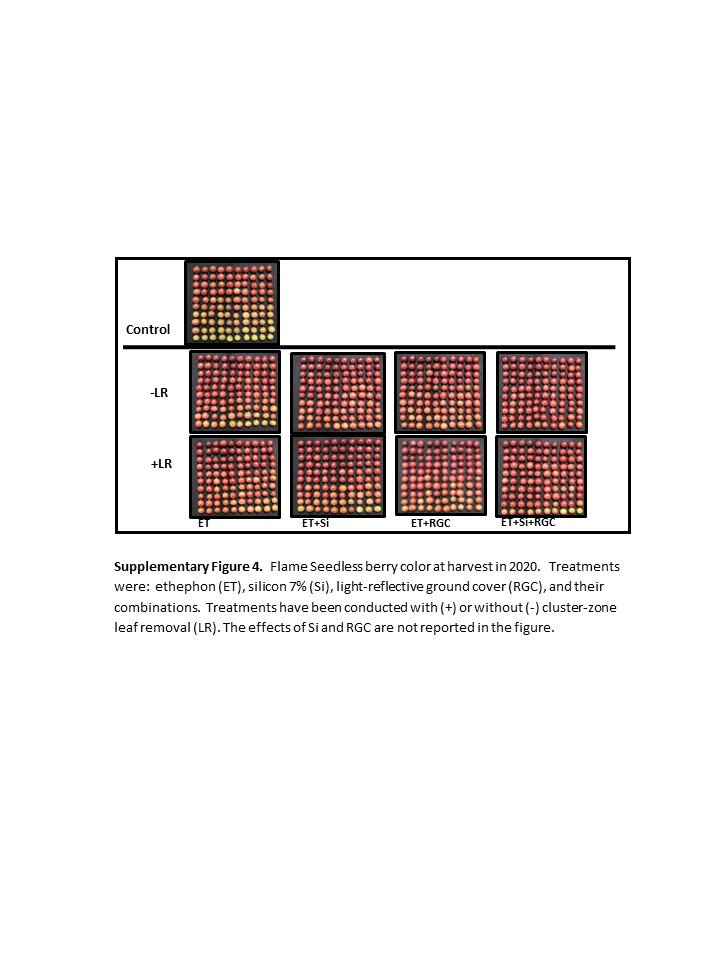

Supplement: Supplementary file 4 [file Image_4.jpeg]

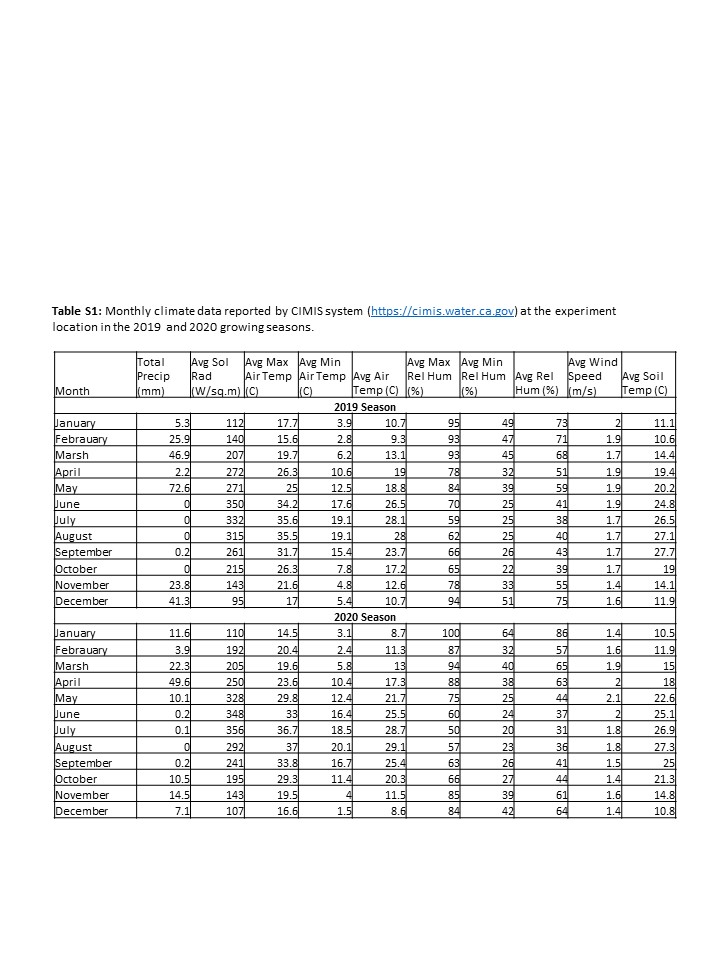

Supplement: Supplementary file 5 [file Image_5.jpeg]
